# Changes in insulin resistance and other metabolic parameters during home quarantine amid the COVID-19 pandemic among the general population

**DOI:** 10.3389/fnut.2025.1610474

**Published:** 2025-10-08

**Authors:** Yuting Zhang, Wenting Chen, Lijian Ran, Jing Wang, Jie Xia, Shilian Li, Qing Mao, Zongtao Chen, Huimin Liu

**Affiliations:** ^1^Health Management Center, Southwest Hospital, Third Military Medical University (Army Medical University), Chongqing, China; ^2^Department of Infectious Diseases, Southwest Hospital, Third Military Medical University (Army Medical University), Chongqing, China; ^3^Chongqing Key Laboratory of Precision Prevention and Control of Viral Infectious Diseases, Chongqing, China

**Keywords:** home quarantine, insulin resistance, triglyceride, glucose, body mass index, metabolic biomarkers

## Abstract

**Background:**

This study aims to investigate the impact of home quarantine on insulin resistance and other metabolic parameters in general populations through the changes in metabolic profile, especially Triglyceride/Glucose (TyG).

**Methods:**

This study included participants who underwent two health checkups at the Health Management Center of the First Affiliated Hospital of the Army Medical University, before and after home quarantine, between December 2021 and February 2023. The home quarantine policy in the Chongqing area was executed between August 2022 and December 2022. Triglyceride/Glucose (TyG), TyG-body mass index (TyG-BMI), and TyG-waist circumference (TyG-WC) were calculated.

**Results:**

A total of 19,957 cases were screened, and 2,473 participants (mean age: 39.35 ± 10.36 years, 69.8% female) were included for the final analysis. Compared to before home quarantine, the TyG (6.80 ± 0.58 vs. 6.83 ± 0.58, *p* < 0.001), TyG-BMI (158.30 ± 31.92 vs. 159.48 ± 31.73, p < 0.001), and TyG-WC (533.53 ± 103.15 vs. 535.78 ± 103.65, *p* = 0.039) increased significantly after the home quarantine. Besides, glucose, lymphocyte, and platelet decreased significantly while BMI, systolic blood pressure, diastolic blood pressure, pulse, total cholesterol, triglyceride, high-density lipoprotein, low-density lipoprotein, uric acid, red blood cell, and monocyte also increased significantly after home quarantine (all *p* < 0.05).

**Conclusion:**

Home quarantine might pose a potential negative impact on insulin resistance risk, indicating that insulin resistance and other metabolic health parameters during the home quarantine period should be monitored regularly.

## Introduction

The pandemic of novel coronavirus pneumonia (COVID-19) has been a global health issue since 2020 (1) to minimize exposure to COVID-19 and avoid the adverse effects of COVID-19 infection. Many countries executed isolation policies including home quarantine, lockdown, travel restrictions, and reducing crowd gatherings. In China, due to the large population, home quarantine was the most commonly used method for self-isolation (2). As a result, China’s home quarantine initiative received positive feedback and was successful in curbing the COVID-19 transmission (2, 3). However, the home quarantine policy also leads to dramatic lifestyle changes during that period. For the majority, the physical activity, eating behaviors, and dietary habits were strictly limited——reduction of outdoor exercise, an increase of sedentary behavior, and unhealthy eating habits (4, 5), which raised significant concerns about the impact on the subjects with the chronic non-communicable disease, such as obesity (6), cardiovascular disease (CVD) (7) and mental health burden (8).

Insulin resistance is a state characterized by reduced responsiveness in insulin-targeting tissues under high insulin levels. It is proven to be associated with many diseases, including metabolic syndrome (9), Type 2 Diabetes Mellitus (T2DM), atherosclerosis, and various metabolic diseases (10). Therefore, the accurate evaluation of insulin resistance is of ultimate importance. Currently, the hyperglycemic clamp is still the gold standard for insulin resistance evaluation. However, due to its inconvinience (11), novel biomarkers including Homeostasis Model Assessment of Insulin Resistance (HOMA-IR) and Triglyceride/Glucose (TyG) Index have been extensively studied in various ethnic populations and shown great predictive value for insulin resistance (12). Recent studies have proven that TyG is superior to HOMA-IR for predicting metabolic syndrome (13). Moreover, Wu et al. revealed that TyG was associated with respiratory diseases like respiratory symptoms and chronic bronchitis, while HOMA-IR did not exhibit the correlation in the same cohort (14).

The accessibility of the TyG index makes it an important tool for the health management of insulin resistance at a population level. Apart from TyG, the TyG-body mass index (TyG-BMI), and TyG-waist circumference (TyG-WC) were other two popular indicators for evaluating insulin resistance. Zhou et al. compared TyG and TyG-BMI, and their results revealed that the TyG index appears to be a more promising indicator for risk stratification (15). TyG-WC was also widely used in the evaluation of insulin resistance like non-alcoholic fatty liver disease (NAFLD) and metabolic-associated fatty liver disease (MAFLD) (16). Several studies have already proven that the COVID-19 lockdown could affect life habits significantly and further contribute to the development of metabolic diseases (17, 18). The pandemic of the Ebola virus, COVID-19 has been controlled, but influenza A and many other viruses still possess great risk potential for public health (19, 20). Simpson et al. also proposed the concept of pathogen X, which could be any pathogen, including but not limited to viruses, bacteria, fungi, parasites, or prions (21). Therefore, home quarantine remains an applicable strategy for epidemic containment during infectious disease outbreaks and requires our further attention. However, to our knowledge, there was no study focused specifically on the change in insulin resistance status based on the hematological examination results and clinical parameters.

Herein, this study aims to investigate the impact of home quarantine measures on metabolic health by examining changes in insulin resistance and related biochemical parameters among the general population.

## Methods

### Study design and population

The home quarantine policy in the Chongqing area of southwestern China was executed between August 2022 and December 2022. Therefore, the before-home quarantine period was defined as December 2021–February 2022, and the after-home quarantine period was defined as December 2022–February 2023 in this study. This retrospective study screened participants who received health checkups at the Health Management Center of the First Affiliated Hospital (Southwest hospital) of the Army Medical University during both before the home quarantine period and after the home quarantine period. The inclusion criteria were: (1) participants > 18 years and < 80 years; (2) participants with at least one health checkup data both before and after the quarantine period. The exclusions were: (1) participants with known history of cardiovascular, cerebrovascular, and metabolic diseases; (2) participants diagnosed with metabolic diseases or a subclinical state of metabolic diseases in the health check-up before home quarantine, including diabetes, fatty liver disease, gout, and hyperthyroidism; (3) pregnant participants; (4) non-native residents; (5) participants with incomplete health check-up data. Personal information was anonymized during the data collection and analysis.

This study was approved by the ethics committee of the Southwest Hospital of Army Medical University, PLA [Approval No. (B)KY2024191]. Due to the retrospective nature of this study, the informed consents were waived by the ethics committee.

### Data collection and outcome definition

The basic information (age, gender), anthropometric data (body weight, height, systolic blood pressure [SBP], diastolic blood pressure [DBP], waist circumference (WC), and hip circumference), and laboratory test were extracted directly from the electronic record. Body mass index (BMI) and Waist-Hip Ratio (WHR) accordingly. The fasting blood sample was obtained for the laboratory examinations, including White blood cells (WBC), Neutrophils (NEU), lymphocytes (LYMPH), Monocytes (MON), Eosinophils (EOS), Total Cholesterol (TC), Triglyceride (TG), High-Density Lipoprotein (HDL), Low-Density Lipoprotein (LDL), fasting blood Glucose (Glu) and Uric Acid (UA). The TyG, TyG-BMI, and TyG-WC indexes were calculated using the following formula (22, 23):


$$\begin{array}{l} \mathrm{TyG}=\ln\;(\;[\text{Fasting triglycerides}\;(\mathrm{mg}/\mathrm{dL})\times \\ \;\;\;\;\;\;\;\;\;\;\;\;\;\;\;\;\;\;\;\;\;\;\;\;\;\;\;\text{Fasting glucose}\;(\mathrm{mg}/\mathrm{dL})]/2\;); \end{array}$$

TyG−BMI=BMI×TyG;

TyG−WC=WC(cm)×TyG.

“*Δ*” represents the growth rate of each index and is calculated as:

Δ = [(Post–home quarantine value − Pre–home quarantine value)/Pre–home quarantine value] × 100%. A Δ value greater than 0 indicates positive growth, while a Δ value less than or equal to 0 indicates negative growth or no growth.

### Statistical analysis

All the statistical analyses were performed using SPSS 26.0 (IBM Corp., New York, USA) and GraphPad Prism 7 (GraphPad Software, California, USA). The distribution of indicators was determined according to the Q-Q plots. Continuous data conformed to the normal distribution were expressed as mean ± standard deviation (SD) and tested by paired sample t-test. Indicators that deviated from the normal distribution were expressed as median (lower quartile, upper quartile) and compared using the paired Wilcoxon signed-rank test. Categorical data was expressed as n (%), and the chi-square test was used for comparison between groups. A two-sided *p* < 0.05 was considered statistically significant in this study.

## Results

### Comparison of characteristics before and after quarantine

**Figure 1:**
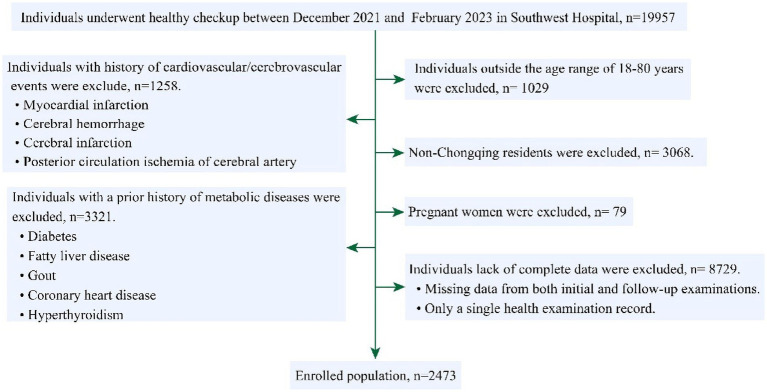
Flowchart of patients screening.

A total of 19,957 cases were screened, among them, after exclusion of patients <18 years or >80 years (*n* = 1,029), non-native residents (*n* = 3,068), with prior cardiovascular/cerebrovascular disease (*n* = 1,258), metabolic diseases (*n* = 3,321), pregnant women (*n* = 79), and incomplete data (*n* = 8,729), 2,473 participants (mean age: 39.35 ± 10.36 years, 69.8% female) were enrolled for final analysis (Figure 1). The average interval between the twice physical checkups was 379.88 ± 38.82 (days).

Compared with the characteristics before quarantine, there were significantly lower Glu (5.38 [5.11, 5.76] vs. 5.53[5.24, 5.88], *p* < 0.001), LYMPH (1.68 [1.39, 2.02] vs. 1.72 [1.40, 2.07], *p* < 0.001), RBC (4.57 ± 0.49 vs. 4.62 ± 0.49, *p* < 0.001), and PLT (219.61 ± 53.11 vs. 223.4 ± 53.2, *p* < 0.001); while higher in BMI (23.20 ± 3.26 vs. 23.14 ± 3.24, *p* = 0.008), SBP (122.63 ± 15.21 vs. 120.66 ± 14.97, *p* < 0.001), DBP (77.16 ± 11.03 vs. 74.99 ± 11.00, *p* < 0.001), Pulse (87.77 ± 12.22 vs. 86.67 ± 12.38, *p* < 0.001), TC (5.07 ± 0.93 vs. 4.78 ± 0.87, *p* < 0.001), TG (0.97 [0.71, 1.44] vs. 0.92 [0.67, 1.35], *p* < 0.001), HDL (1.52 ± 0.32 vs. 1.46 ± 0.32, *p* < 0.001), LDL (3.09 ± 0.67 vs. 2.92 ± 0.67, *p* < 0.001), UA (319.56 ± 79.97 vs. 311.65 ± 80.15, *p* < 0.001), MON (0.31 [0.25, 0.38] vs. 0.29 [0.24, 0.36], *p* < 0.001) (Table 1).

**Table 1:** Characteristics of participants before and after home quarantine.

Index	Before quarantine (*n* = 2,473)	After quarantine (*n* = 2,473)	*p*-value
Weight (Kg)	60.87 ± 11.00	60.96 ± 11.02	0.125^a^
BMI (kg/m^2^)	23.14 ± 3.24	23.20 ± 3.26	0.008^a^
WC (cm)	77.99 ± 10.01	77.93 ± 10.17	0.631^a^
HC (cm)	95.42 ± 6.02	95.29 ± 6.16	0.117^a^
WHR	0.82 ± 0.07	0.82 ± 0.07	0.646^a^
SBP (mmHg)	120.66 ± 14.97	122.63 ± 15.21	<0.001^a^
DBP (mmHg)	74.99 ± 11.00	77.16 ± 11.03	<0.001^a^
Pulse (BPM)	86.67 ± 12.38	87.77 ± 12.22	<0.001^a^
TC (mmo/L)	4.78 ± 0.87	5.07 ± 0.93	<0.001^a^
TG (mmo/L)	0.92 (0.67, 1.35)	0.97 (0.71, 1.44)	<0.001^b^
HDL (mmo/L)	1.46 ± 0.32	1.52 ± 0.32	<0.001^a^
LDL (mmo/L)	2.92 ± 0.67	3.09 ± 0.67	<0.001^a^
Glu (mmo/L)	5.53 (5.24, 5.88)	5.38(5.11, 5.76)	<0.001^b^
UA (μmo/L)	311.65 ± 80.15	319.56 ± 79.97	<0.001^a^
WBC (10^9^/L)	5.40 (4.64, 6.42)	5.44 (4.63, 6.39)	0.337^b^
NEU (10^9^/L)	3.20 (2.59, 3.91)	3.24 (2.62, 3.95)	0.338^b^
LYMPH (10^9^/L)	1.72 (1.40, 2.07)	1.68 (1.39, 2.02)	<0.001^b^
MON (10^9^/L)	0.29 (0.24, 0.36)	0.31 (0.25, 0.38)	<0.001^b^
RBC (10^12^/L)	4.62 ± 0.49	4.57 ± 0.49	<0.001^a^
HGB (g/L)	140.29 ± 15.76	140.18 ± 15.56	0.505^a^
PLT (10^9^/L)	223.4 ± 53.2	219.61 ± 53.11	<0.001^a^

TC, Total Cholesterol; HDL, High-Density Lipoprotein, LDL, Low-Density Lipoprotein, WC: Waist Circumference; HC, Hip Circumference.

a Paired sample t-test.

b Wilcoxon rank-sum test.

### Changes in metabolic parameters

Statistical analysis revealed significant post-quarantine elevations across three metabolic indices: the TyG demonstrated an increment from 6.80 ± 0.58 to 6.83 ± 0.58 (*p* < 0.001), TyG-BMI increased from 158.30 ± 31.92 to 159.48 ± 31.73 (*p* < 0.001), and TyG-WC showed progression from 533.53 ± 103.15 to 535.78 ± 103.65 (*p* = 0.039) (Table 2).

**Table 2:** Comparison of metabolic indexes before and after home quarantine.

Metabolic indexes	Before quarantine (*n* = 2,473)	After quarantine (*n* = 2,473)	*p* value
TyG	6.80 ± 0.58	6.83 ± 0.58	<0.001^a^
TyG-BMI	158.30 ± 31.92	159.48 ± 31.73	<0.001^a^
TyG-WC	533.53 ± 103.15	535.78 ± 103.65	0.039^a^

^a^Paired sample *t*-test.

### Subgroup analysis of TyG changes

**Figure 2:**
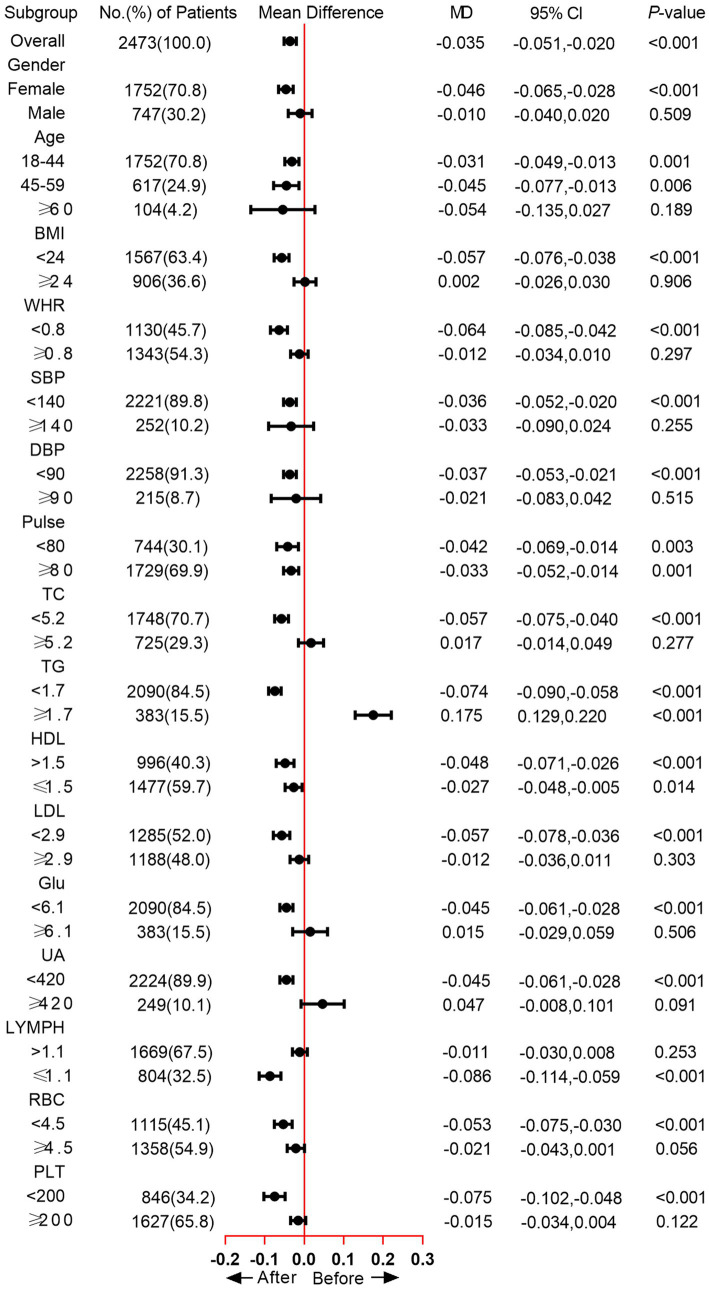
Change of TyG across different subgroups.

The subgroup analysis showed that the TyG was significantly increased in the female subgroup (mean difference [MD] = −0.046, *p* < 0.001), while no significant difference was observed in male participants. As for Age subgroups, significantly increased TyG was found in participants between 18 and 44 (MD = −0.031, *p* = 0.001) and 45–59 (MD = −0.045, *p* = 0.006). In the BMI < 24 subgroup, the MD of TyG was −0.057, and the *p* value was < 0.001. WHR < 0.8 group also exhibited increased TyG after home quarantine (MD = −0.064, *p* < 0.001). Moreover, we also analyzed the change of TyG in blood pressure, TC, TG, HDL, LDL, Glu, UA, LYMPH, RBC, and PLT subgroups, except for the TG ≥ 1.7 subgroup, an increased or unchanged TyG was observed in all other subgroups (Figure 2). When comparing the positive growth percentage of indicators, there were significant differences in PLT, HGB, NEU, UA, LDL, TC, and BMI between genders (Supplementary Figure 1).

## Discussion

The present study demonstrated that home quarantine was associated with significant elevations in insulin resistance indices, specifically the TyG index, TyG-BMI, and TyG-WC. Furthermore, distinct variation patterns were observed in other metabolic parameters. These findings collectively suggest that quarantine measures may exert substantial effects on physiological homeostasis, particularly regarding insulin resistance characteristics.

The home quarantine significantly altered lifestyles, characterized by reduced physical activity, increased sedentary behavior, and unhealthy eating habits for the majority of individuals (4, 5). Changes in dietary structure, physical inactivity, and psychological pressure may explain weight gain and metabolic impairment. Fowler revealed that relative physical activity level was associated with HOMA-IR (24). Eriksson et al. demonstrated that psychological distress was associated with insulin resistance, significantly higher HOMA-IR was observed in patients with psychological distress (25), Robinson showed that metabolic syndrome and psychological burden may have independent effects on non-communicable diseases risk (26), although no previous study directly focused on psychological status and insulin resistance during the COVID-19 lockdown, many studies revealed that psychological stress was increased during the quarantine period, which could contribute to the worsening of insulin resistance indices (27, 28). Elevated psychological stress can activate the hypothalamic–pituitary–adrenal (HPA) axis and sympathetic nervous system, leading to increased cortisol and catecholamine secretion, which in turn impairs insulin signaling, reduces glucose uptake in peripheral tissues, and promotes hepatic gluconeogenesis—mechanisms known to exacerbate insulin resistance (29, 30). In addition, stress-related behavioral changes, such as reduced physical activity, altered sleep patterns, and unhealthy dietary habits, may further aggravate insulin resistance (30, 31).

While a study from Poland found that reduced intake of vegetables, fruits, and legumes during the epidemic blockade, increased their intake of meat, dairy products, and fast food, leading to weight gain (32). Moreover, Pinto revealed that excessive and prolonged sedentary behaviors can lead to insulin resistance and other diseases. A more direct evidence was reported by Gonzalez et al., they revealed a worse insulin resistance status after the lockdown period (33). Besides, other studies demonstrated that the disruption of lifestyle and unhealthy behaviors altered circadian biology and exposed people to health problems such as weight gain/obesity (34), dyslipidaemia (35), and cardiometabolic health (36), which have been proven to be associated with metabolic abnormalities (37).

TyG, TyG-BMI, and TyG-WC as novel metabolic parameters have received extensive research focus. The relation between TyG-related parameters and COVID-19 infection was also found in several previous studies. Ren et al. reported an increased risk of COVID-19 severity and morbidity and higher TyG (38). Another study performed by Chang et al. showed that a high TyG index was associated with an increased risk for severe COVID-19 complications (39). For the TyG-BMI and TyG-WC, although the relevant reports were much less compared to the TyG index, Shabestari still showed a positive association between the TyG-BMI index and the risk of COVID-19 (40). The current evidence collectively underscores that dysregulated TyG indices, including TyG-BMI and TyG-WC, demonstrate consistent associations with adverse COVID-19 outcomes. Notably, while COVID-19 infection status was not systematically documented in our cohort, the ubiquitous nature of viral exposure during the study period may contribute to observed TyG elevations through indirect pathophysiological mechanisms. This investigation provides novel insights by identifying quantifiable increases in TyG parameters, specifically during home quarantine—a previously unexamined temporal window in metabolic research. Interestingly, we observed that HDL increased after home quarantine, which was in alignment with the findings of Ojo et al. (41), while both Valenzise and Bonfrate reported that HDL level decreased after the lockdown period, especially in females (6, 42), this might be related to changes in diet or lifestyle during the quarantine period. However, this interesting finding should be further confirmed in other population-based studies.

The results of our study also showed a lower level of LYMPH, EOS, and BAS in the general population after long-term isolation at home. This finding is in correlation with a previous study, patients with COVID-19 in China showed lower lymphocyte counts, higher leukocyte counts, and neutrophil-lymphocyte ratio (NLR), as well as lower percentages of MON, EOS, and BAS (43). This evidence revealed the potential association between blood cell count change and SARS-COV-2 infection. We further found elevated UA, BMI, TC, LDL, and Glu levels during the quarantine. More importantly, similar findings were present in children and adolescents, Morelli et al. showed that COVID-19 lockdown had a negative impact on adolescents’ metabolic and inflammatory profile (44), while Dong et al. analyzed the metabolic health of Chinese children and concluded that long-term lockdown due to COVID-19 outbreak might cause adverse impact on children’s metabolism and lead to increased risk of cardiovascular diseases (45).

Still, this study has certain limitations. First, although the sample size is sufficient, this is a single-center study, which may restrict the generalizability of the results to other regions or healthcare settings in China. Large-scale validation is required for future clinical applications. Second, limited parameters were included in this study. The dietary habits, daily activity information, and other insulin resistance-related parameters (e.g., HOMA-IR) were not recorded in this study. Third, all the participants underwent home quarantine, no control participants were included in this study. Fourth, the absence of COVID-19 infection data represents an additional limitation. In this retrospective design, the before–home quarantine period was defined as December 2021 to February 2022 to match the post-quarantine period and reduce seasonal bias; however, this may introduce potential selection bias and omit changes occurring between March and July 2022. Finally, due to the retrospective nature of this study, we did not survey the diet habits of the participants, we acknowledged that diet habits could affect the metabolic status, and in the future prospective study, we would take these factors into consideration.

Home quarantine may exacerbate insulin resistance risk, highlighting the necessity for regular monitoring of metabolic parameters (e.g., insulin resistance indices, and glycemic control markers) during isolation periods. This clinical vigilance could facilitate timely interventions to mitigate progression towards diabetes mellitus and cardiovascular complications, particularly in individuals with pre-existing metabolic vulnerabilities.

## Data Availability

The original contributions presented in the study are included in the article/Supplementary material, further inquiries can be directed to the corresponding authors.
