# Supplementary figures and images for: Changes in insulin resistance and other metabolic parameters during home quarantine amid the COVID-19 pandemic among the general population
Source: Front Nutr. 2025 Oct 8;12:1610474. doi: 10.3389/fnut.2025.1610474 (PMC12540093; doi:10.3389/fnut.2025.1610474)

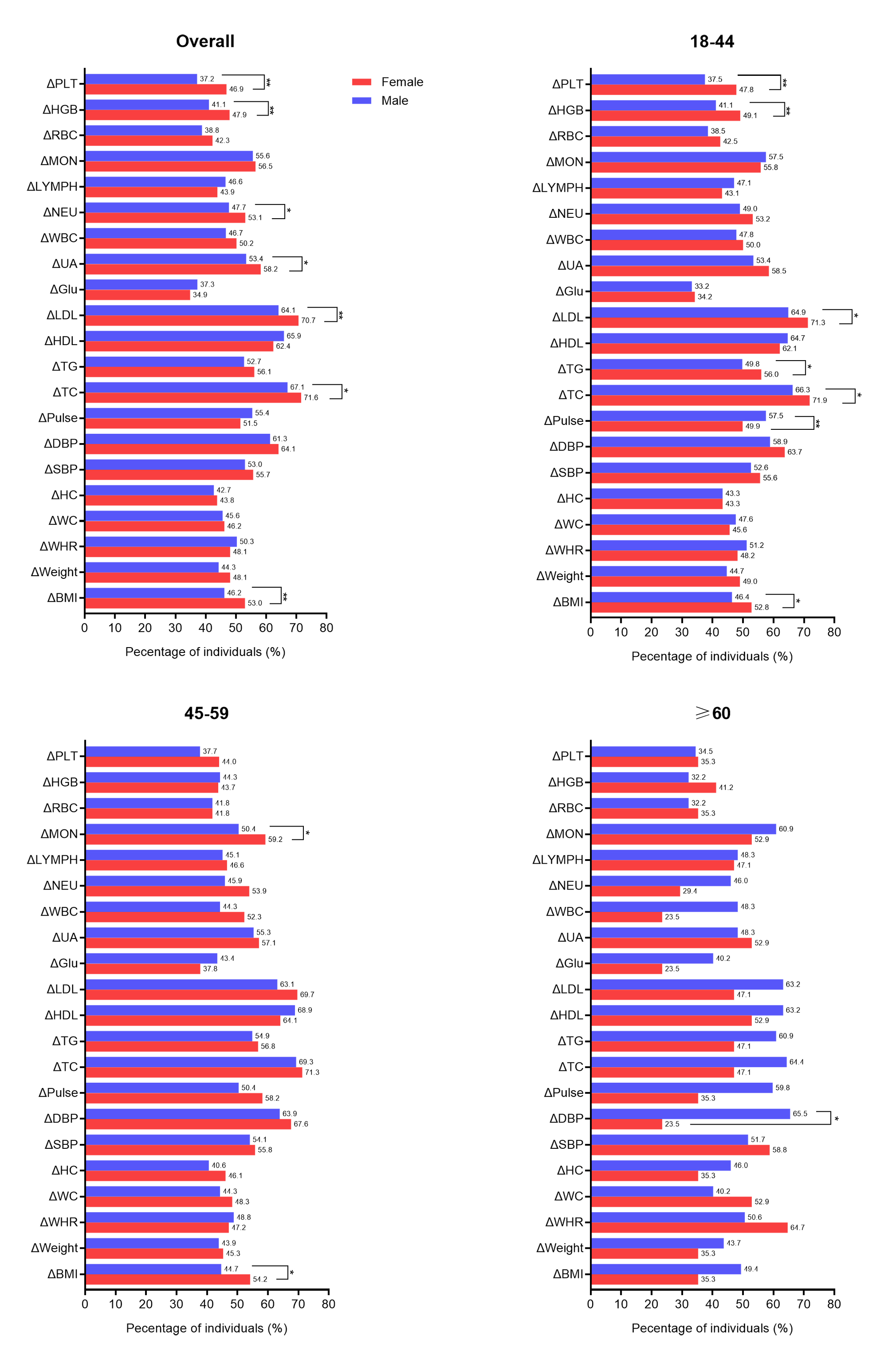

Supplement: Supplementary file 1 [file Image_1.JPEG]
